# Burnout during the COVID-19 pandemic among nurses in Taiwan: the parental role effect on burnout

**DOI:** 10.1186/s12913-024-11159-w

**Published:** 2024-06-04

**Authors:** Yong-Hsin Chen, Mohsen Saffari, Chung-Ying Lin, Hsiu-Mei Tang, Ching-wen Yang, Chiu-Hsian Lee, Wei-Yao Wang, Gwo-Ping Jong

**Affiliations:** 1 Department of Public Health, Chung Shan Medical University, Taichung, 402 Taiwan; 2 Department of Occupational Safety and Health, Chung Shan Medical University Hospital, Taichung, 402 Taiwan; 3 Health Research Center, Life Style Institute, Baqiyatallah University of Medical Sciences, Tehran, Iran; 4 Health Education Department, Faculty of Health, Baqiyatallah University of Medical Sciences, Tehran, Iran; 5 Institute of Allied Health Sciences, College of Medicine, National Cheng Kung University, Tainan , Taiwan; 6 Department of Nursing, Chung Shan Medical University, No. 110, Sec. 1, Jianguo N. Rd., South Dist, Taichung, 402306 Taiwan; 7 Department of Nursing, Chung Shan Medical University Hospital, No. 110, Sec. 1, Jianguo N. Rd., South Dist, Taichung, 402306 Taiwan; 8 Department of Internal Medicine, Chung Shan Medical University Hospital, No. 110, Sec. 1, Jianguo N. Rd., South Dist, Taichung, 402306 Taiwan, ROC; 9 School of Medicine, Chung Shan Medical University, Taichung, Taiwan, ROC

**Keywords:** Nurses, Client burnout, Parental role, Leisure activity

## Abstract

**Background:**

During the COVID-19 pandemic, medical workers were concerned about the care of their children or family members and the impact of being separated from them. This increased stress could harm the relationship between nurses and patients. This study assessed how medical workers’ parental role may affect burnout during such a high-stress period.

**Methods:**

This cross-sectional observational study was carried out in 2021 during the COVID-19 pandemic. The client burnout (CB) scale of the Copenhagen Burnout Inventory, the Nordic Musculoskeletal Questionnaire, and a demographic questionnaire were used. Statistical methods such as the t-test, one-way ANOVA, and univariable/multiple linear regression were applied.

**Results:**

A total of 612 nurses were included in this study. The likely risk factors of CB were identified and the parenthood effect was found to be associated with reduced CB. The parental role and leisure activity with family and friends on CB were found to have an impact. Engaging in leisure activity with family and playing the role of a parent diligently will help relieve nurses’ burnout from frequent contact with patients and their families, thus lowering the risk of clinical burnout.

**Conclusion:**

The parental role, family/friends relationships, and a complex work environment associated with nurses’ burnout during the COVID-19 pandemic. This finding allows us to re-examine the importance of family life and parent–child relationships in high-stress work environments.

**Supplementary Information:**

The online version contains supplementary material available at 10.1186/s12913-024-11159-w.

## Introduction

Burnout was first described in 1974 by the clinical psychologist Herbert Freudenberger, who defines symptoms of burnout as including malaise, fatigue, frustration, cynicism, and inefficacy [1]. One major cause of burnout is related to people’s employment. Hence, burnout had been recognized as an “occupational phenomenon” resulting from chronic workplace stress that has not been managed [2]. Clinical burnout has been widely recognized by symptoms such as emotional exhaustion, physical fatigue, cognitive impairment, disturbed sleep, and functional impairment [3, 4] and could lead to depression, or anxiety disorders [3]. Past studies suggested that overtime [5], shift work [6], lack of sleep [6], and chronic diseases [7] are the primary reasons for burnout. In addition, musculoskeletal pain could be associated with mental health issues such as burnout [8]. Fortunately, good exercise habits [9] helped reduce burnout levels. Moreover, participating in leisure activities may be another potential protector for nurses because it relatively improves people’s physical and mental health [10] and also helps reduce role conflict [11], enhances employees’ satisfaction with life [12], and addresses negative emotions [13], etc.

Of note, burnout is common among healthcare providers. Previous studies, for example, regardless of differences in geographical regions and specialties, the prevalence of burnout for nurses ranged from 10.51 to 33% worldwide [14, 15]. Burnout not only affects nurses’ well-being [16] but also has negative impacts on the work force [17]. For instance, 31.5% of nurses who resign cited burnout as a reason in USA [18]. Nurses have to interact frequently and directly with patients and their families in all healthcare processes [19]. In addition, the reduced satisfaction and safety of patients would easily contribute to increased emotional exhaustion and subsequent burnout for nurses [20, 21].

Fortunately, getting support from their family may effectively minimize burnout feelings [22]. Thus, family support is considered vital for addressing burnout [23]. Nevertheless, the parental role is both complex and stressful and causes parents to experience exposure to chronic parenting stress [24].

During the COVID-19 pandemic, hospital workers, especially nurses, from Taiwan revealed their fears concerning taking care of children or family members and the stress of being separated from family [25]. These double factors of stress and fear could cause conflicts in the relationship between nurses and patients. Consequently, burnout among nurses is likely to increase so it is important to understand how the parental role relate to burnout resulted from patients and their family. Specifically, burnout might be worsened for nurses by exhaustion from taking care of children and patients simultaneously. Interestingly, the extent to which experiences in one role act to improve the quality of life in the other role works according to the theory of work–family enrichment [26]. Moreover, the family leisure involvement was the strongest predictor of family satisfaction from the parent perspective [12]. Therefore, whether did nurses who play parental role and positively engage leisure activity with family and friends may really reduce burnout from being in frequent contact with patients and patients’ family members? That is a worth issue further explored. Based on this, the present study proposed two hypotheses for further verifying:

### Hypothesis 1

*The parental role is related to burnout from being in frequent contact with patients and patients’ family members for nurses.*

### Hypothesis 2

*Positively engaging leisure activity with family and friends in free time is a mediating factor that parental role reduce burnout from being in frequent contact with patients and patients’ family members for nurses.*

This result will let us rethink the importance of the parent-child relationship in work under high-stress conditions that will be helpful for medical institutions formulating a more extensive strategy of health promotion combined family living in nurses.

## Materials and methods

### Participants and procedures

This cross-sectional observational study was conducted during the COVID-19 pandemic between March and April 2021 in a hospital affiliated with a medical university in Taichung, Taiwan. Taiwan reported its first imported COVID-19 case in January 2020 and experienced its initial local outbreak in April 2021 [27]. Registered nurses (including nursing administrators) were approached to complete a survey. All 612 nurses who had served for one year in the hospital received a QR code by email linking to Google Forms questionnaires, of which 512 (83.66%) were deemed valid after excluding those with missing data. The survey included the validated questionnaires of the Copenhagen Burnout Inventory, the Nordic Musculoskeletal Questionnaire, and a background information sheet asking about demographics, family, living habits, work, and physical health. The study protocol was approved by the institutional review board of Chung Shan Medical University (No: CS1-21108).

### Measures

**Demographic information.** We assessed the participants’ age, marital status (“Yes” or “No”), parenthood (“Yes” or “No”), service department (Anesthesiology, Emergency, Hemodialysis, Intensive Care Unit, Outpatients, Operating Room, Ward, or Others), overtime pattern (More than 80 h per month, between 45 h and 80 h, less than 45 h, or seldom), work schedules (shift, night, or day), alcohol drinking habit in the past month (every day, occasionally, or never), having a regular exercise habit (at least once per day, at least once weekly, at least once per month, less than once per month, or never), Engaging in leisure activity with family and friends (LAFF) in free time. According to a suitable for parametric tests 5-point Likert scale method [28], the response options were “always”, “often”, “sometimes”, “seldom”, or “never” were scored as 100, 75, 50, 25, and 0 points, respectively; the points indicated the LAFF level. The response options of chronic diseases were “Yes” or “No”.

**Burnout.** We used the Chinese version of the Copenhagen Burnout Inventory (CBI). The CBI is reliable and valid for the assessment of burnout problems [29]. Specifically, the CBI consists of three subscales including personal burnout, work-related burnout, and client burnout (CB). Of the three, the CB subscale was adopted to measure burnout resulted from being in frequent contact with patients and patients’ family members for the present study. All 13 items for the CB subscale are listed in supplementary information Table S1. The response options—“always,” “often,” “sometimes,” “rarely,” and “never/almost never”—were scored as 100, 75, 50, 25, and 0 points, respectively; the calculated mean values indicated the CB level. A higher score indicates a higher level of CB.

**Musculoskeletal discomfort.** The Nordic Musculoskeletal Questionnaire which has been modified and translated by the Taiwan Institute of Occupational Safety and Health [30] was adopted to determine the frequency and sites of pain among the participants. The options of frequency of every site of pain were every day, once a week, once a month, once every six months, or at least once every six months, corresponding to 100, 80, 60, 40, and 20 points, respectively. A higher score on the Nordic Musculoskeletal Questionnaire indicates a higher level of musculoskeletal discomfort. Factor analysis [31] was adopted to determine new underlying variables that could effectively explain the questionnaire. These new variables by factor analysis would be adjusted variables in a linear regression model of burnout and parental role. The results of the factor analysis are presented in Supplementary Information Table S2.

### Data analysis

Regarding the procedures of statistical analyses, we adopted some steps determined the effect of parental role on CB level.

Step 1. The t test and One-way ANOVA were adopted testing the difference on CB level among two or multiple independent variables.

Step 2. Pearson correlation analysis was adopted determined the association in statistical terms between the continuous variables and CB level.

Step 3. We adopted the multiple linear regression determining the independent risk or protective factors for CB level in the presence of adjusted variables.

Step 4. We adopted mediation analysis determining the mediating factor of survey variable on CB level. Mediation effects were analyzed using the strategy proposed by Baron and Kenny [32], in which (1) the independent variable significantly affects the mediating factor (first-stage effect), (2) the independent variable significantly affects the dependent variable in the absence of the mediating factor (only recommended but not required [33]), (3) the mediating factor exerts a significant unique effect on the dependent variable (second-stage effect), and (4) the effect of the independent variable on the dependent variable weakens upon the addition of a mediator to the model. The formulas are as follows:

$$Y={b}_{01}+cX$$

$$M={b}_{02}+aX$$

$$Y={b}_{03}+{c}^{{\prime }}X+bM$$

where *X* is an independent variable, $Y$ is a dependent variable, $M$ is the mediating factor, *a* is the linear regression coefficient of *X* against mediating factor, *b* is the linear regression coefficient of mediating factor against $Y$, *c* is the linear regression coefficient of *X* against $Y$, and *c’* is the linear regression coefficient of *X* against $Y$ with mediating factor as the adjusting variable. The standard errors of *a* and *b* are represented by *s*_*a*_ and *s*_*b*_, respectively. The formula of the Sobel test is as follows:

$$Z=\frac{a\times b}{\sqrt{{b}^{2}{{s}_{a}}^{2}+{a}^{2}{{s}_{b}}^{2}}}$$

The results exceeding |1.96|, |2.57|, and |3.90| (for a two-tailed test) are significant at α = 0.05, 0.01, and 0.0001, respectively.

The mediation proportion (MP) is defined as the dimensionless proportion of the effect of an independent variable on a dependent variable mediated through the mediating factor, whose formula is as follows [34]:

$$MP=\frac{a\times b}{c{\prime }+a\times b}$$

Analyses were performed using SAS Enterprise Guide 6.1 software (SAS Institute Inc., Cary, NC, USA), and the results were deemed statistically significant at *P* < 0.05.

## Results

Regarding the description of basic variables of nurses in Table 1, among them, the lower quartile, median, and higher quartile of age were 27, 35, and 43 years and the mean age was 35.33 ± 9.30 years. Nursing staff are mainly composed of female (96.48%). Slightly less than half of the participants were married (41.41%). More than one-third (35.74%) had children. Results showed that the majority of participants worked in general hospital wards (32.42%), Outpatients (14.84%), and the Intensive Care Unit (13.67%). 41.4% of the participants reported a sleep duration of six hours daily or less. The proportion of individuals who seldom experienced overtime in the past month was 55.66%. Work schedules were classified as working shift rotation, night, and day shift, whose proportions were 34.77%, 21.48%, and 43.75%, respectively. The proportion of individuals who never drank alcohol in the past month was 63.48%. Those who had a weekly exercise habit were 45.12%. The proportion of individuals who “always” and “often” engaged in LAFF in their free time were 4.69% (*N* = 24) and 24.80% (*N* = 127), respectively. Of the participants, 195 (38%) reported that they suffer from a chronic disease.

**Table 1:** Sample characteristics

Survey Variables	Individuals (*N* = 512)	proportion (%)/mean ± SD
***Age*** (mean = 35.33 ± 9.30)		
Less than 27	152	29.69
Between 27 and 35	117	22.85
Between 35 and 43	122	23.83
more than 43	121	23.63
***Sex***		
male	18	3.52
female	494	96.48
***Marital status***		
married	212	41.41
single	300	58.59
***Parenthood***		
Yes	183	35.74
No	329	64.26
***Service department***		
Anesthesiology	20	3.91
Emergency	26	5.08
Hemodialysis	23	4.49
Intensive Care Unit	70	13.67
Outpatients	76	14.84
Operating room	38	7.42
Ward	166	32.42
Other	93	18.16
***Sleep duration per day***		
Less than 5 h	21	4.10
Between 5 h and 6 h	191	37.30
Between 6 h and 7 h	212	41.41
Between 7 h and 8 h	69	13.48
More than 8 h	19	3.71
***Overtime state***		
More than 80 h per month	1	0.20
Between 45 h and 80 h	20	3.91
Less than 45 h	206	40.23
Seldom work overtime	285	55.66
***Work schedules***		
Working shift rotation	178	34.77
Night	110	21.48
Day	224	43.75
***Alcohol drinking habit in the past month***		
Every day	1	0.20
Occasionally	186	36.33
Never	325	63.48
***Exercise habit***		
At least once per day	32	6.25
At least once weekly	199	38.87
At least once per month	110	21.48
less than once per month	132	25.78
Never	39	7.62
***Engaging in LAFF in free time***		
Always (100)	24	4.69
Often (75)	127	24.80
Sometimes (50)	270	52.73
Seldom (25)	88	17.19
Never (0)	3	0.59
***The presence of chronic diseases***		
Yes	195	38.09
No	317	61.91

N = individuals; LAFF = leisure activity with family and friends

Table 2 demonstrates the difference on CB level among two or multiple independent variables. The age categories indicated a significant difference on CB level (*P* = 0.003). The lowest CB level was in individuals whose ages were over 43 years (mean = 28.96 ± 16.49). The individuals who were parents reported low CB (mean = 30.76 ± 17.59; *P* = 0.009) compared to those who had no children. There were significant differences of CB level among nurses who work in different service departments (*P* = 0.001). Among them, nurses who worked in hemodialysis section reported the highest CB level (mean = 40.94 ± 20.05) and those who worked in Operating room reported lowest (mean = 25.22 ± 14.17) CB level, respectively.

The individuals whose sleep duration per day was less than 6 h reported higher CB level (mean = 35.71 ± 17.44, *P* = 0.016) than those who reported sleep duration per day over 6 h. Experienced overtime sustained high CB level (mean = 36.29 ± 17.82, *P* = 0.001). Similarly, the individuals who worked rotational shifts experienced higher CB (mean = 35.58 ± 17.46, *P* = 0.049) than those who worked either a day or night shift. Table 2 demonstrated that the participants who drank alcohol in the past month sustained high CB (mean = 36.72 ± 18.47, *P* = 0.002). Nurses with chronic disease also reported high CB (mean = 35.83 ± 19.06, *P* = 0.018).

**Table 2:** The difference on CB level among two or multiple survey variables

		CB level	
Survey Variables	Individuals	mean (SD)	*P*
***Age***			
Less than 27	152	34.07 (18.92) ^a^	0.003
Between 27 and 35	117	33.33 (17.26) ^a^	
Between 35 and 43	122	37.36 (16.53) ^a^	
More than 43	121	28.96 (16.49) ^b^	
***Marital status***			
Yes	212	31.84 (16.87)	0.077
No	300	34.64 (18.08)	
***Parenthood***			
Yes	183	30.76 (17.59)	0.009
No	329	34.99 (17.48)	
***Service department***			
Anesthesiology	20	26.25 (19.45) ^cd^	0.001
Emergency	26	36.54 (20.53) ^ab^	
Hemodialysis	23	40.94 (20.05) ^a^	
Intensive Care Unit	70	33.93 (18.81) ^abc^	
Outpatients	76	35.25 (16.42) ^ab^	
Operating room	38	25.22 (14.17) ^d^	
Ward	166	35.62 (17.38) ^ab^	
Others	93	30.11 (15.80) ^bcd^	
***Sleep duration per day***			
Less than 6 h per day	212	35.71 (17.44)	0.016
More than 6 h per day	300	31.90 (17.61)	
***Overtime pattern***			
Experienced overtime	227	36.29 (17.82)	0.001
Seldom worked overtime	285	31.42 (17.17)	
***Shift***			
Yes	178	35.58 (17.46)	0.049
No	334	32.36 (17.63)	
***Alcohol drinking in the past month***			
Yes	187	36.72 (18.47)	0.002
No	325	31.62 (16.86)	
***Weekly regular exercise***			
Yes	231	31.85 (18.78)	0.062
No	281	34.82 (16.52)	
***The presence of chronic diseases***			
Yes	195	35.83 (19.06)	0.018
No	317	32.03 (16.54)	

SD = standard deviation; *P* = *p*-value; ^a, b,c, d^, Means with the same letter are not significantly different (by Duncan’s multiple-range test)

According to Supplementary information Table S2, the relatively large factor loading values for Factors 1 and 2 corresponded to the pain sites of the neck, shoulders, and ankles, respectively. Based on this, we defined Factors 1 and 2 as Pain in Neck and Both Shoulders and Pain in Both Ankles. Table 3 demonstrates that CB level was associated with LAFF level (*r* = -0.154, *P* = 0.001), musculoskeletal pain in the neck and both shoulders (*r* = 0.17, *P* < 0.0001) and both ankles (*r* = 0.09, *P* = 0.042).

**Table 3:** Correlations between continuous variable and CB

	CB	
	r	*P*
LAFF level	-0.15	0.001
Pain in Neck and Both Shoulders	0.17	< 0.0001
Pain in Both Ankles	0.09	0.042

r = Pearson correlation coefficient; *P* = *p*-value

According to Tables 2 and 3, Age, parenthood, service department, sleep duration per day, overtime pattern, shift, alcohol drinking in the past month, the presence of chronic diseases, LAFF level, pain in neck and both shoulders, and pain in both ankles were confounders of increased CB. They would been added to multiple linear regression model against CB. Table 4 demonstrated parenthood (B = -4.17, *P* = 0.02) and LAFF level (B = -0.12, *P* = 0.001) was independent protective factors of increased CB in the presence of adjusted variables.

**Table 4:** Multiple linear regression of survey variable against CB

	CB level (*N* = 512)		
Survey Variable	B	β	*P*
***Parenthood***			
Yes vs. No	-4.17	-0.11	0.022
***Engaging in LAFF in free time***			
LAFF level	-0.12	-0.14	0.001

N = individuals; B = the linear regression coefficient of the parental role against CB; β = the standardized linear regression coefficient of the parental role against CB; *P* = *p*-value; ^a^, the model’s adjusted age, service department, sleep duration per day, overtime state, shift-working, alcohol drinking in the past month, the presence of chronic diseases, pain in the neck and both shoulders, and pain in both ankles.

**Fig. 1:**
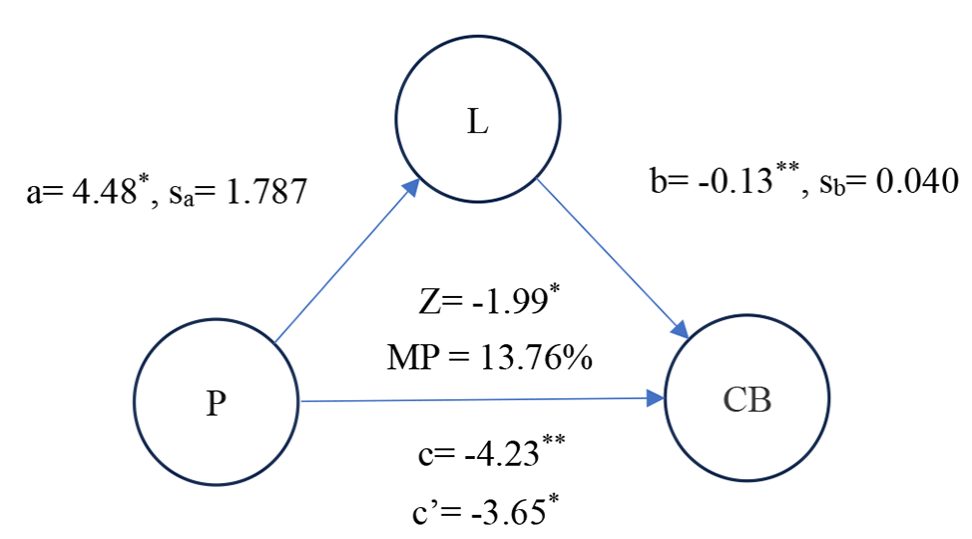
Mediation model on the relationship between parental role and CB; ^*^P < 0.05; ^**^P < 0.01; L = LAFF level; *P* = parent’s role

Figure 1 presented a simple mediation model. Among them, First-stage effect: the parental role effect on increased LAFF level was significant (a = 4.48, *P* < 0.05). Second–stage effect, increased LAFF level (mediating factor) exerts a significant effect on reduced CB level (b = -0.13, *P* < 0.01). The effect of parenthood on CB level weakens upon the addition of LAFF level (mediating factor) to the univariate linear regression model (c = -4.23, c’ = -3.65). Moreover, we confirmed the mediating model was significant in statistic through Sobel test (Z = -1.99, *P* < 0.05), too. So, the model of Fig. 1 demonstrated increased LAFF level was a mediating factor that parental role effect weakens CB. In addition, the mediation effect of LAFF was partial mediation whose MP was 13.76%.

## Discussion

The present study determined that the sleep duration less than 6 h per day (mean = 35.71 ± 17.44, *P* = 0.016), experienced overtime (mean = 36.29 ± 17.82, *P* = 0.001), working shift rotation (35.58 ± 17.46, *P* = 0.049), alcohol drinking in the past month (mean = 36.72 ± 18.47, *P* = 0.002), pain in neck and both shoulders (*r* = 0.17, *P* < 0.0001)/both ankles (*r* = 0.09, *P* = 0.042), and the presence of chronic diseases (mean = 35.83 ± 19.06, *P* = 0.018) were significantly associated with increased CB level. These reasons for burnout have been confirmed by previous studies. For instance, insufficient sleep (< 6 h) [35] and disturbed sleep [36] were identified as the main risk factors for burnout development. Notably, work-related factors such as overtime [37] and shift rotation [38] were also associated with increased burnout. In addition, whether alcohol reduces stress is debatable [39]. Evidence reveals that alcohol abuse or dependence among surgeons is associated with burnout development [40]. Moreover, one’s physical health condition could affect burnout. For instance, the onset of localized neck/shoulder and/or lower back pain [41–43] and chronic diseases [44]were associated with burnout symptoms.

Despite hospital workers during the COVID-19 pandemic revealed that fears related to taking care of children and being separated from family [25], the present study found that nurses who play a parental role still sustain a lower CB level than those who do not play a parental role in the presence of adjusted confounders (Table 4, B = -4.17, *P* = 0.022). In addition, we also found that individuals who play a parental role sustained high LAFF level than those who do not play a parental role in the presence of adjusted confounders (Table 4B = -0.12, *P* = 0.001). A prior study for Burnout Syndrome during the COVID-19 pandemic in Serbia demonstrated the level of burnout for women decreases with more children [45]. Moreover, a previous study has shown that work and family identities and support from the family were individual, work, and family antecedents of enrichment [46]. Notably, participation in family leisure could improve interactions, cohesion within families, family functioning, and satisfaction [47] and further increase parental and children’s well-being [48]. The previous study demonstrated that low levels of social support from family and friends, and a lack of leisure time were associated with an increased risk for parental burnout [49]. The previous and our research all demonstrated that individuals who engage in leisure activities will keep well mental health including burnout. Moreover, the prior study also directly indicated the family involvement was closely related to family satisfaction from parent perspective [50]. So, we adopted the positivity of engaging in leisure activities with family and friends in free time as a proxy indicator that parent if positively involve family living. We through mediation analysis of Fig. 1 found positively engaging in leisure activity plays a mediating factor between parental role and weaken CB (Z = -1.99, *P* < 0.05). It means positively involve family living really enhance the quality of nurse’s role during high stress period that is consistent with the theory of work–family enrichment [26] - participation in one role may enrich the quality of life in the other role. Based on above findings, we confirm Hypothesis 1 and 2 in Introduction. The two results construct a framework about family relationship and reduced burnout; individuals who feel family well-being could enhance another work-related role that could be helpful for improving client burnout for nurses. This study clearly delineates the roles and operational mechanism of the work-family enrichment theory during the COVID-19 pandemic - aspects that were rarely examined in previous research.

However, LAFF partially mediate effect (MP = 13.76%) that parental role weakens CB, that hints LAFF is only one of reasons of effect. Therefore, we must adopt new questionnaire explored other possible reasons about family and burnout in further research.

Although those who completed the burnout questionnaire and reported high burnout level are not necessarily at risk for clinical burnout [51], people with short-term stress show an elevated level of burnout [52] and a quicker recovery, which is a more favorable prognosis compared with those with clinical burnout [53]. Therefore, the Copenhagen Burnout Inventory is an effective early warning tool for clinical burnout. Due to the mediation model of an observational study could be biased [54], the causal relationship among parental role, LAFF, and burnout is higher risk of judgement. Therefore, we avoid using the sentence “causal relationship” in our study’s result. In addition, we think that the study adopted LAFF as a proxy indicator for enhanced parental roles is insufficient despite of the trend that positively engaging in leisure strength enhanced parental role will not change. The future research should use more rigorous and suitable questionnaire measured parent burnout or satisfaction of family living. It is noteworthy that we did not collect age about raising child and investigate whether the participants are the primary caregivers for children or other family members who can assist with childcare responsibilities. We think they could affect parent stress of family living and could be a confounder of burnout.

## Conclusion

Insufficient sleep per day (less than 6 h per day), overtime work, working shift rotation, alcohol drinking in the past month, and chronic pain are related to increased burnout from being in frequent contact with patients and patients’ family members for nurses. Nurses who positively engage in leisure activity in free time could weaken burnout resulted from client. In addition, engaging in leisure activity with family in free time is one of important reasons that nurses who are parent sustain low client burnout than those who are not parent. This finding also allows us to re-examine the importance of family life and parent–child relationships in high-stress work environments. So, medical institutions should supply more resources and courses or lectures to help nurses how to play the successful parent role and encourage nurses who raise children into more healthy leisure activities in their free time with children.

### Electronic supplementary material

Below is the link to the electronic supplementary material.

Supplementary Material 1

## Data Availability

Datasets used and/or analyzed during the current study are available from the corresponding author on reasonable request.
