# Supplementary material for: Burnout during the COVID-19 pandemic among nurses in Taiwan: the parental role effect on burnout
Source: BMC Health Serv Res. 2024 Jun 4;24:703. doi: 10.1186/s12913-024-11159-w (PMC11151642; doi:10.1186/s12913-024-11159-w)
Supplement: Supplementary file 1 — Supplementary Material 1 [file 12913_2024_11159_MOESM1_ESM.docx]

**Supplementary information Table S1** The 6 items for the Client Burnout scale

| item | Items for Client Burnout, are as follows: |
| --- | --- |
| 1 | Do you find it hard to work with clients? |
| 2 | Does it drain your energy to work with clients? |
| 3 | Do you find it frustrating to work with clients? |
| 4 | Do you feel that you give more than you get back when you work with clients? |
| 5 | Are you tired of working with clients? |
| 6 | Do you sometimes wonder how long you will be able to continue working with clients? |

Supplementary information Table S2 MS pain sites and factor analysis of the NMQ

| MS pain sites | N | % | Frequency score | Factor loading^1^ | |
| --- | --- | --- | --- | --- | --- |
|  |  |  | Mean (SD) | Factor 1 | Factor 2 |
| Neck^a^ | 195 |  | 73.53(20.16) | **0.30** | 0.01 |
| Left shoulder^a^ | 125 |  | 73.44(23.11) | **0.34** | 0.00 |
| Right shoulder^a^ | 124 |  | 78.55(20.59) | **0.36** | 0.03 |
| Upper back | 93 |  | 76.77(18.72) | 0.14 | -0.03 |
| Waist or lower back | 194 |  | 71.65(22.36) | 0.10 | -0.02 |
| Left elbow | 29 |  | 77.24(19.07) | -0.04 | -0.03 |
| Right elbow | 36 |  | 82.22(21.26) | -0.05 | -0.04 |
| Left wrist | 38 |  | 78.42(23.88) | -0.04 | -0.04 |
| Right wrist | 63 |  | 75.24(25.83) | -0.07 | -0.08 |
| Left hip/thigh/buttock | 24 |  | 70.83(22.83) | -0.04 | -0.09 |
| Right hip/thigh/buttock | 25 |  | 72.80(24.41) | -0.02 | -0.05 |
| Left knee | 31 |  | 72.90(17.55) | -0.06 | -0.09 |
| Right knee | 36 |  | 73.33(21.38) | -0.01 | -0.07 |
| Left ankle^b^ | 14 |  | 65.71(33.66) | -0.05 | **0.51** |
| Right ankle^b^ | 12 |  | 70.00(32.47) | -0.01 | **0.60** |
|  |  | Eigenvalues | | 4.98 | 1.62 |
|  |  | Explained variation % | | 58.81 | 19.18 |

^1^, All numbers are standardized scoring coefficients, N, individuals, SD, standard deviation; ^a^, Factor 1 correspond pain sites; ^b^, Factor 2 correspond pain sites.
